# Value of Delta Fractional Flow Reserve (ΔFFR) For Predicting Coronary Ischemic Lesions

**DOI:** 10.31661/gmj.v9i0.1528

**Published:** 2020-10-03

**Authors:** Vahid Eslami, Morteza Safi, Mohammad hasan Namazi, Mehdi Pishgahi, Amir Eftekharzade, Sayyed Ali Eftekharzadeh

**Affiliations:** ^1^Shahid Beheshti University of Medical Sciences, Cardiovascular Research Center, Tehran, Iran

**Keywords:** Fractional Flow Reserve, Coronary Artery Diseases, Myocardial Infarction, Stenosis

## Abstract

**Background:**

The decrease in fractional flow reserve (FFR) after adenosine administration from baseline FFR value (termed as ΔFFR) may reflect the compensatory capacity of the microvascular circulation and thus may predict significant coronary stenotic lesions. We aimed to investigate whether baseline FFR and ΔFFR can help identify the coronary ischemic lesion and its severity.

**Materials and Methods:**

This cross-sectional study was performed on 154 consecutive patients (Mean age 62.42 ± 9.36 years) that underwent coronary angiography and with definitive intermediate coronary lesions at any of the coronary vessels. FFR was calculated by dividing the mean distal intracoronary pressure by the mean arterial pressure. ΔFFR was also defined as the difference between baseline FFR and hyperemic FFR (considering FFR<0.75 as the criteria for ischemia).

**Results:**

The area under receiver-operating characteristic curve for baseline FFR was found as 0.933, and for ΔFFR was 0.946 indicated high values of both indices for predicting ischemic lesions. The best cut-off point for baseline FFR and ΔFFR for discriminating ischemic lesions from the normal condition was 89.5 (yielding a sensitivity of 92.2% and a specificity of 68.0%) and 9.5 (yielding a sensitivity of 96.0% and a specificity of 85.3%), respectively.

**Conclusion:**

Our study could successfully demonstrate the high value of both baseline FFR and ΔFFR for predicting coronary ischemic lesions with the cut-off values of <89.5 and >9.5, respectively.

## Introduction

The assessment of the physiological aspects of coronary arteries at the time of coronary angiography is beneficial to quantify the severity of arterial lesions leading to cardiac ischemic events. This goal can now be achieved by measuring some physiological parameters, such as the fractional flow reserve (FFR) [1,2]. FFR is defined as the ratio of maximal achievable blood flow in a coronary artery in the presence of stenosis to the hypothetical maximal achievable blood flow in that same epicardial artery in the absence of the stenosis [3]. Because this parameter is not affected by any changes in the microcirculation status or hemodynamic instability [4], it can be used with high confidence for the identification of ischemia-producing lesions in the coronary arteries and thus for assessment of the severity of cardiac ischemia. However, various cut-off values have been introduced for FFR to discover cardiac ischemia. As shown by Pijls *et al*. [5], an FFR value of less than 0.75 was associated with reversible ischemia on noninvasive stress testing with high sensitivity and specificity [5]. In other words, a value of less than 0.75 can be considered as the gold cut-off value for predicting cardiac ischemia [6-8]. A cut-off point higher than 0.80 was shown to completely rule out the presence of cardiac ischemia [9,10]. According to the Society for Cardiovascular Angiography and Interventions guideline and based on the value of FFR, the coronary ischemic lesions are categorized as non-ischemic stenosis (FFR> 0.8), ischemia-producing stenosis (FFR<0.75), and a gray zone with FFR values between 0.75 and 0.80 [11]. In this regard, FFR> 0.75 is now acceptable as a good index for the need for revascularization [12]. The value of FFR is mainly affected by diffuse coronary artery involvement, left ventricular hypertrophy, and especially small vessel disease [13,14]. The dilatation capacity of the microvascular circulation can potentially affect the value of FFR. In other words, the studies have shown that the decrease in FFR after adenosine administration from baseline FFR value (termed as ΔFFR) reflects the compensatory capacity of the microvascular circulation in patients with the significant coronary stenotic lesion [15]. Although pure FFR is now accepted as a reliable index for defining the functional significance of the coronary lesions, the meaning of ΔFFR remains unclear. We aimed to investigate whether ΔFFR can help identify the coronary ischemic lesion and its severity.

## Materials and Methods

 This cross-sectional study was performed on 154 consecutive patients who underwent coronary angiography and with definitive intermediate coronary lesions (50 to 60% stenosis) at any of the coronary vessels in Modarres and Nikan hospitals in Tehran during 2017-2019.

 All the procedures and protocols of this study were approved (code: IR.SBMU.MSP.REC.1398.369) by the Ethical Committee of Shahid Beheshti University of Medical Sciences. Those subjects with previous coronary revascularization, any evidences of systemic infections, and inflammatory disorders were all excluded from the study. The patients were divided into three groups, according to the FFR (min, hyperemic) results. The patients with a FFR value greater than 0.80 were included in group I (n=115), the patients with a FFR value between 0.75 and 0.80 were included in group II (n=14), and the patients with a FFR value less than 0.75 were included in group III (n=25). All the patients were received anti-coagulated with at least 5000 units of unfractionated heparin. A Radi 0,014 XT PW pressure-monitoring guidewire (St. Jude Medical, USA) introduced through a 6–8 guiding catheter and calibrated. Then the pressure guidewire advanced through the coronary artery until it was positioned at the distal of the stenosis. After recording baseline distal intracoronary pressure, intracoronary adenosine (20–150g bolus) was administered to induce maximal vasodilatation. The minimum distal coronary pressure was recorded. FFR was calculated by dividing the mean distal intracoronary pressure by the mean arterial pressure. This procedure was repeated two times; then, minimum FFR calculation was used to determine the severity of the lesion. The lesion was accepted significant if FFR<0.75 after adenosine administration (FFR min, hyperemic). The following formula calculated FFR from measurements of FFR at baseline conditions (FFR base, resting) and after adenosine administration (FFR min, hyperemic):

 FFR (FFR base – FFR min) × 102.

 The study endpoint was to assess the relationship between ΔFFR and baseline FFR (before injecting adenosine) with the presence of ischemic lesions.

###  Statistical Analysis

 Descriptive analysis was used to describe the data, including mean ± standard deviation (SD) for quantitative variables and frequency (percentage) for categorical variables. Chi-square test, independent t-test, and Mann-Whitney U test, as well as multivariable logistic regression, were used for comparison of variables. To assess the value of baseline FFR and ΔFFR to predict ischemic lesion, the receiver-operating characteristic (ROC) curve analysis was employed. For the statistical analysis, the statistical software IBM SPSS Statistics for Windows version 22.0 (IBM Corp. Released 2013, Armonk, New York) was used. P-value <0.05 were considered as significant differences.

## Results

**Figure 1:**
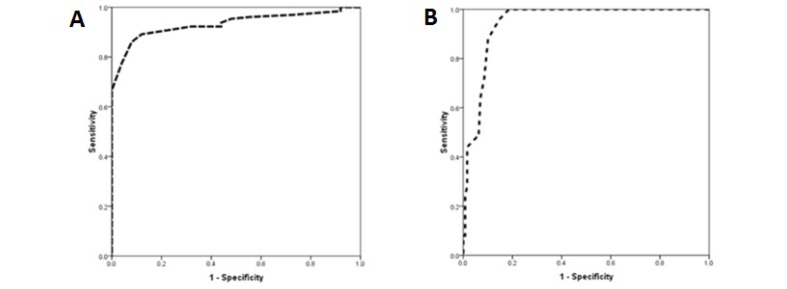
Area under the ROC curve for baseline FFR (A) and ΔFFR (B) for predicting coronary ischemic lesions.

Table-1 shows the baseline characteristics, angiographic measurements, and FFR results of the patients in different FFR groups. The baseline characteristics and the angiographic features of the patients were similar except for more prevalence of left ventricular dysfunction, smoking, and family history of coronary diseases in those with FFR ≤0.8. The multivariable logistic regression analysis (Table-2) and considering FFR<0.75 as the criteria for ischemia, the main determinant of ischemia were ΔFFR (P˂0.001), left ventricular dysfunction (P=0.023), and family history of coronary diseases (P=0.047). The value of both baseline FFR and ΔFFR to detect the ischemic lesion, which was defined according to the FFR<0.75 were evaluated by ROC analysis. The area under ROC curve for baseline FFR was 0.933 (95% confidence interval [CI]: 0.893–0.983, P<0.001, Figure-1A) and for ΔFFR was 0.946 (95% CI: 0.913–0.980, P<0.001, Figure-1B) indicating high value of both indices for predicting ischemic lesions. The best cut-off point for baseline FFR and ΔFFR for discriminating ischemic lesions from the normal condition was 89.5 (yielding a sensitivity of 92.2% and a specificity of 68.0%) and 9.5 (yielding a sensitivity of 96.0% and a specificity of 85.3%), respectively.

## Discussion

In various studies, baseline FFR introduced as a good marker for predicting the cardiac functional significance of ischemic lesions, especially in the intermediate state, so the patients with FFR less than 0.75 can be candidates for coronary revascularization and those with FFR higher than 0.80 is basically free from ischemic lesions. However, the correct interpretation of the lesion and decide revascularization in those who categorized as the gray zone remains difficult. It is now hypothesized that ΔFFR as the difference between FFR base (resting) and FFR-min (hyperemic) can be an indicator for the compensatory response capacity of the vascular bed to the significantly obstructive lesion [15]. Our study demonstrated that the high value of both baseline FFR and ΔFFR for discriminating ischemic lesions with high sensitivity and acceptable specificity. The best cut-off values for both parameters were <89.5 and >9.5, respectively. Therefore, considering these two parameters can also be very useful to determine the ischemic status in the group in the gray zone category. In this regard, the cut-off value achieved for ΔFFR seems to be more highly predictive for ischemia. In total, it seems that considering both cut-off values can discriminate ischemia from normal conditions with considerably higher precision. In other words, those patients with baseline FFR<89.5 and ΔFFR>9.5 have severe ischemic lesions and can certainly be for coronary revascularization. A few similar studies could obtain similar findings on the value of baseline FFR and ΔFFR for predicting ischemic lesions. As similarly shown by Kocaman *et al*. [15], when ≥15 is accepted as the cut-off value for ΔFFR, the specificity and sensitivity were 95% and 59%, respectively. However, the sensitivity obtained by the authors was significantly low, and therefore it seems that lower cut-off points (as revealed in our study) are needed to provide both sensitivity and specificity. However, contrarily, in Xaplanteris *et al*. study [16], although baseline FFR could predict future clinically significant lesions (with the area under the ROC curve of 0.736), the predicting value of ΔFFR was not proved so that worsening FFR and improving FFR based on the ΔFFR values were observed only in 25% and 8% of affected patients, respectively. The ΔFFR parameter not only can be used for assessing the severity of ischemic lesions but also it can be considered as a good index for following-up the response to revascularization procedures. Because ΔFFR can determine the clinical condition in the group categorized in the gray zone or intermediate ischemia, and due to this fact that a majority of such patients are planned for percutaneous coronary intervention (PCI), the improvement in ΔFFR after PCI can be a good indicator for procedural success [17,18]. Thus, determining ΔFFR can identify the extent and location of stenosis; help in appropriate revascularization protocol and stent implantation, as well as predict the postoperative outcome.

## Conclusion

 Our study could reveal the high value of both baseline FFR and ΔFFR for predicting coronary ischemic lesions with the cut-off values of <89.5 and >9.5, respectively. Because of the uncertainty of those in the gray zone of FFR (0.75 to 0.80), both pointed parameters can be effectively used for deciding for medical therapy or revascularization procedure. In this regard, the assessment of ΔFFR is even more sensitive and specific in predicting severe ischemic lesions.

## Conflict of Interest

 Authors declare there are was no any conflict of interest.

**Table 1:** Clinical and Laboratory Parameters in the FFR Groups. Data Are Presented as n (%) Or Mean±SD.

**Variables**	**FFR groups**			**P-value**
	**<0.75**	**0.75 – 0.80**	**>0.80**	
**Male gender**	11 (44.0)	8(57.1)	69 (60.0)	0.342
**Age, year**	64.52 ± 8.78	61.55 ± 9.43	65.86 ± 8.91	0.125
**Age>60 years**	16 (64.0)	9 (64.3)	65 (56.5)	0.708
**LVEF<50%**	23 (95.8)	12 (85.7)	28 (24.3)	<0.001
**Type of ACS**				
Unstable angina	20 (80.0)	10 (71.4)	99 (86.1)	0.319
NSTEMI	5 (20.0)	4 (28.6)	16 (13.9)	
**Coronary vessels involved**				
LAD	12 (48.0)	8 (57.1)	74 (64.3)	0.645
LCX	7 (28.0)	3 (21.4)	15 (13.0)	
RCA	6 (24.0)	3 (21.4)	15 (13.0)	
LM	0 (0.0)	0 (0.0)	2 (1.7)	
Diagonal	0 (0.0)	0 (0.0)	5 (4.3)	
OM	0 (0.0)	0 (0.0)	2 (1.7)	
PDA	0 (0.0)	0 (0.0)	2 (1.7)	
**Site of coronary involvement**				
Distal	0 (0.0)	0 (0.0)	3 (2.6)	0.718
Mid	18 (72.0)	10 (71.4)	72 (62.6)	
Ostium	0 (0.0)	1 (7.1)	3 (2.6)	
Proximal	7 (28.0)	3 (21.4)	37 (32.2)	
**Medical history**				
Hypertension	14 (56.0)	7 (50.0)	92 (80.0)	0.006
Hyperlipidemia	11 (44.0)	5 (35.7)	79 (68.7)	0.005
Diabetes mellitus	13 (52.0)	9 (64.3)	61 (53.0)	0.712
Smoking	11 (44.0)	6 (42.9)	20 (17.4)	0.004
Family history of CAD	11 (44.0)	3 (21.4)	16 (13.9)	0.003
**FFR measurement**				
Baseline FFR	85.96 ± 3.36	88.14 ± 3.94	95.53 ± 3.81	<0.001
Secondary FFR	72.64 ± 1.49	76.14 ± 1.65	91.02 ± 4.67	<0.001
ΔFFR	13.32 ± 3.40	12.00 ± 3.80	4.51 ± 2.33	<0.001

**LVEF: **Left ventricular ejection fractions, ** ACS: **Acute coronary syndrome, **NSTEMI:** Non-ST-elevation myocardial infarction, **LAD: **Left anterior descending, **LCX: **Left circumflex, ** RCA:** Right coronary artery, **LM: **Left main, ** OM: **Obtuse marginal, **PDA: **Patent ductus arteriosus, **CAD: **Coronary artery disease, **FFR:** Fractional flow reserve

**Table 2:** Multivariable Logistic Regression Model to Determine Main Predictors for Ischemic Lesions

**Variables**	**B**	**S.E.**	**Wald**	**P-value**	**OR**
**ΔFFR**	-0.497	0.105	22.390	<0.001	0.609
**Sex**	-0.620	0.758	0.670	0.413	0.538
**Age**	-0.065	0.044	2.206	0.137	0.937
**LVEF**	-0.136	0.060	5.183	0.023	0.873
**Type of ACS**	-0.580	0.911	0.405	0.525	0.560
**Coronary vessel involved**	-0.187	0.353	0.281	0.596	0.829
**Hypertension**	-0.479	0.770	0.387	0.534	0.619
**Hyperlipidemia**	-0.202	0.770	0.068	0.794	0.817
**Diabetes mellitus**	-0.780	0.785	0.988	0.320	0.458
**Smoking**	0.644	0.765	0.709	0.400	1.905
**Family history**	1.817	0.820	4.911	0.027	6.151

**FFR**: Fractional flow reserve, **LVEF**: Left ventricular ejection fractions, **ACS**: Acute coronary syndrome, **OR**: Odds ratio
